# The Other Side

**Published:** 1893-01-07

**Authors:** 


### PASSING TOPICS.

### THE OTHER SIDE.

IF it be really true that a recently deceased inhabitant of the antipodes, who had succeeded in making his "pile," bequeathed several thousands of pounds to a village hospital subject to a charge of one shilling weekly payable to his widow, we can only express a hope that the whole of the story is not comprised within these narrow limits. We would fain believe it will turn out that the shilling a week is only supplementary to an equitable and substantial provision for the one who ought to have been the subject of the testator's first consideration.

Yet, as the experience of many charity workers has shown, there is upon rare occasions a disposition, more or less accentuated, to give undue preference to public as compared with personal claims, and it is not impossible that our Australian friend may have elected to furnish in his own act a signal illustration of the lengths to which human nature will sometimes go in perversity. It is often our task to protest against the neglect our philanthropic institutions suffer at the hands of the very rich, but we should respond with all the alacrity in the world to the plea that the claims of kindred are stronger than those of the most valuable charitable agency, and that families are not to be impoverished in order that institutions, however useful, may flourish.

Most of those whose experience of charity finance is sufficiently complete are able to recall some legacy or another which has been accepted with embarrassment, and with a sincere desire that a refusal were possible. That, of course, the law forbids, but we cannot help believing that justice and humanity would alike be served by an amendment of the law designed to prevent a wilful overlooking of blood relations. Upon the one hand we wish cordially it could be enacted that no large property should pass to heirs without paying tribute to the poor, but not the less cordially do we regret that it is impossible now for the best and purest work of benevolence to be saved from occasionally becoming an instrument for the exercise of spite or criminal negligence.

Similar convictions force themselves upon us concerning another remarkable bequest recorded within the past few days. A lady described as of "independent means" has had, so it is said, the sum of £150,000 bequeathed to her by an old gentleman, a perfect stranger, whom she had casually aided by procuring for him a glass of cold water when he was suffering from faintness. We have no wish to cavil at the truly gigantic proportions gratitude, a somewhat rare ornament of character, has assumed upon this occasion. But for all that, we encourage a pious aspiration that the old gentleman's wealth has not been alienated upon so slender a pretext from his natural heirs, and we cannot help thinking that it would well become the lucky legatee were she to take steps to satisfy herself before entering into possession that she was not asserting merely legal as distinguished from moral rights, to the injury of those who have a righteous claim to the inheritance. If her benefactor had no needy relations, well and good; in the other case, and failing restitution, we should heartily regret that the law is far too ready to pronounce for the sanity of a testator who perhaps, in respect of the very act which brings his affairs within the cognizance of the law, may have given the most convincing proof of his inability to exercise a sound judgment. AGRICOLA.

